# Molecular characterization and biofilm-formation analysis of *Listeria monocytogenes*, *Salmonella* spp., and *Escherichia coli* isolated from Brazilian swine slaughterhouses

**DOI:** 10.1371/journal.pone.0274636

**Published:** 2022-09-20

**Authors:** Rebecca Lavarini dos Santos, Emilia Fernanda Agostinho Davanzo, Joana Marchesini Palma, Virgílio Hipólito de Lemos Castro, Hayanna Maria Boaventura da Costa, Bruno Stéfano Lima Dallago, Simone Perecmanis, Ângela Patrícia Santana

**Affiliations:** Faculty of Agronomy and Veterinary Medicine, University of Brasília (UnB), Brasília, Federal District, Brazil; Cornell University, UNITED STATES

## Abstract

This study aimed to verify the presence of *Listeria monocytogenes*, *Salmonella* spp., and *Escherichia coli* in two Brazilian swine slaughterhouses, as well as to perform antibiograms, detect virulence and antimicrobial resistance genes, and evaluate the *in vitro* biofilm-forming capability of bacterial isolates from these environments. One *Salmonella* Typhi isolate and 21 *E*. *coli* isolates were detected, while *L*. *monocytogenes* was not detected. *S*. Typhi was isolated from the carcass cooling chamber’s floor, resistant to several antimicrobials, including nalidixic acid, cefazolin, chloramphenicol, doxycycline, streptomycin, gentamicin, tetracycline, and sulfonamide, and contained resistance genes, such as *tet(B)*, *tet(C)*, *tet(M)*, and *ampC*. It also showed moderate biofilm-forming capacity at 37°C after incubating for 72 h. The prevalence of the 21 *E*. *coli* isolates was also the highest on the carcass cooling chamber floor (three of the four samplings [75%]). The *E*. *coli* isolates were resistant to 12 of the 13 tested antimicrobials, and none showed sensitivity to chloramphenicol, an antimicrobial prohibited in animal feed since 2003 in Brazil. The resistance genes *MCR-1*, *MCR-3*, *sul1*, *ampC*, *clmA*, *cat1*, *tet(A)*, *tet(B)*, and *blaSHV*, as well as the virulence genes *stx-1*, *hlyA*, *eae*, *tir α*, *tir β*, *tir γ*, and *saa* were detected in the *E*. *coli* isolates. Moreover, 5 (23.8%) and 15 (71.4%) *E*. *coli* isolates presented strong and moderate biofilm-forming capacity, respectively. In general, the biofilm-forming capacity increased after incubating for 72 h at 10°C. The biofilm-forming capacity was the lowest after incubating for 24 h at 37°C. Due to the presence of resistance and virulence genes, multi-antimicrobial resistance, and biofilm-forming capacity, the results of this study suggest a risk to the public health as these pathogens are associated with foodborne diseases, which emphasizes the hazard of resistance gene propagation in the environment.

## Introduction

*Escherichia coli*, *Salmonella* spp., and *Listeria monocytogenes* are among the main bacteria involved in foodborne diseases, and have been evaluated in depth to prevent future outbreaks across the world [1,2].

*Salmonella* spp. is mostly involved in foodborne illnesses worldwide [3]. Approximately 2,500 *Salmonella* serotypes have been identified, the majority of which may adapt to several animal hosts, including humans [4]. According to the Epidemiological Profile of Etiological Agents published by the Brazilian Ministry of Health [5], *E*. *coli* is the second most common bacterial agent involved in food poisoning outbreaks in Brazil. In addition, this bacterium also causes foodborne outbreaks worldwide and its presence indicates fecal contamination [6,7].

Furthermore, the persistence of foodborne pathogens in biofilms has also been reported, mostly on food contact surfaces, affecting product quality, quantity, and safety [8]. In the meat industry, bacterial biofilms are a major concern due to accumulation in areas difficult to sanitize, leading to cross-contamination and food spoilage [9–11]. In food processing units, *Listeria* spp. has been detected on equipment surfaces, impermeable sealing substances, conveyor belts, and drains, persisting in the industrial environment from months to years [12]. Moreover, *Listeria* spp. can grow at 4–10°C, which is the temperature range commonly used to control food infections, and can become a problem during food handling [13,14].

The presence of these pathogenic microorganisms is a safety hazard to food industries, since they are unlikely to be eliminated from the processing line due to their proliferation and possible biofilm formation [12,15], thus increasing resistance to sanitizers as well as physical and chemical treatments [9,16]. In addition to compromising food hygiene and posing a public health risk, antibiotic resistance and gene transfer among bacteria are associated, potentially increasing the number of circulating virulent strains [17–19].

Since Brazil is the fourth largest pork exporter, and a good performance in this market is due to competitive prices coupled with quality products, it is essential to pay attention to pathogenic microorganisms that can lead to sanitary crises or represent barriers to commercialization [20]. Estimating the number of foodborne outbreaks related to pork meat is difficult due to the lack of reliable data; the contamination rate is under-reported as the majority of cases are not registered [21].

Meanwhile, there have been few reports of biofilms in Brazilian pork industries and there is an absence of data in the Federal District of Brazil and the surrounding region. This study aimed to detect *E*. *coli*, *Salmonella* spp., and *L*. *monocytogenes* in the environment and equipment of swine slaughterhouses in the Federal District of Brazil. Molecular characterization and antimicrobial resistance testing of strains isolated from biofilms were also conducted.

## Material and methods

### Origin of the samples

Samples were collected from two swine slaughterhouses (A and B) located in the Federal District of Brazil and two visits were made to each swine slaughterhouse between 2019 and 2021, with a minimum 24 h interval. Swabbing (Absorve®; São Paulo, Brazil) of a delimited area was used for sampling the surfaces, equipment, and utensils [22]. A total of 44 swab samples were collected from 11 points each of two slaughterhouses (A and B) during two visits, using one swab per point per visit. The sample points were defined according to the protocols presented by Cabral *et al*. [23], Nicolau & Bolocan [24], and Barros *et al*. [22]divided into facilities (floors, walls, and drains) and equipment/utensils (tables, bleeding knife, dehairing machine, and carcass splitting saw).

Samples were collected between the last post-slaughter hygiene process at the end of the workday and before starting the daily activities with pre-slaughter hygiene procedures, due to the relation of bacterial permanence on surfaces post hygiene in industries with the presence of bacterial biofilms [25,26].

### *Salmonella* spp., *L*. *monocytogenes*, and *E*. *coli* isolation

*E*. *coli* was isolated from the swab samples and identified using a previously described methodology [27]. Briefly, the swabs were transferred from tubes containing 0.1% peptone water (HiMedia®; Mumbai, India) to tubes containing 9 mL 1% buffered peptone water (Acumedia®; Melbourne, Australia) and incubated at 37°C for 24 h. Subsequently, they were streaked onto Eosin Methylene Blue agar plates and incubated at 37°C for 24 h to observe the growth of typical *E*. *coli* colonies (blue-black colonies with or without metallic green reflex). The *E*. *coli* colonies were subjected to standard biochemical tests for microbial identification [28,29].

For *L*. *monocytogenes* isolation, swab samples were analyzed according to the methodology described by the Brazilian Normative Instruction n°40 [30] for research and microbial *L*. *monocytogenes* isolation. The surface swabs were transferred from tubes containing 0.1% peptone water to tubes containing 9 mL 1% buffered peptone water and incubated at 37°C for 24 h. After incubation, 1 mL culture was transferred to 9 mL UVM broth (Acumedia®) and incubated at 35°C for 24 h. Then, 0.1 mL culture was transferred to 10 mL Fraser broth (Acumedia®) and incubated at 35°C for 24 h. Fraser broth tubes with observed esculin hydrolysis were plated on MOX agar plates (Difco™; Berkshire, England) and incubated at 35°C for 24 h. Small colonies with a halo of esculin hydrolysis were collected, transferred to Brain Heart Infusion (BHI) broth (Difco™), and incubated at 37°C for 24 h. A turbidity test was performed using droplets obtained from the BHI broth. Gram staining and catalase tests were performed [31].

To identify *Salmonella* spp., swab samples were analyzed according to the protocols described in the Technical Manual for Laboratory Diagnosis of *Salmonella* spp. [32] and ISO 6579/2002 [33]. Briefly, the surface swabs were transferred from tubes containing 0.1% peptone water to tubes containing 9 mL 1% buffered peptone water and incubated at 37°C for 24 h. After incubation, 1 and 0.1 mL cultures were transferred to 10 mL Selenite cystine broth (Merck®; DarmstadtGermany) and Rappaport Vassiliadis broth (Fluka™; Buchs, Germany), respectively, and incubated at 42°C for 24 h. Next, the above-mentioned broths were streaked onto selective modified Brilliant-green Phenol-red Lactose Sucrose agar plates (Acumedia®) and incubated at 37°C for 24 h. Three colonies with morphological characteristics of *Salmonella* spp. were streaked on Triple Sugar Iron (Acumedia®) agar slants and incubated at 37°C for 18–24 h. TSI tubes with potential *Salmonella* growth were biochemically tested as indicated in the Technical Manual for Laboratory Diagnosis of *Salmonella* spp. [32], including urea hydrolysis, phenylalanine deaminase, indole production, Voges–Proskauer test, methyl red test, lysine decarboxylase, and citrate utilization. Positive controls for standardization were provided by the Oswaldo Cruz Foundation, Rio de Janeiro.

Colony PCR analysis [34] was performed to identify and confirm *Salmonella* spp. and amplification reactions were performed in a final volume of 25 μL, containing 2 Units Taq DNA polymerase (Invitrogen®. Waltham, MA, USA), 2 mM phosphate deoxyribonucleotides (Invitrogen®), 1× buffer (200 mM Tris-HCl, pH 8.4; 500 mM KCl; Invitrogen®), 1.5 mM MgCl_2_ (Invitrogen®), and 1 μM primers. The reaction was performed in a MyCycler thermal cycler (BioRad®; Hercules, CA, USA) under the following conditions: initial denaturation at 94°C for 3 min, 35 cycles at 94°C for 0.40 min, annealing temperature according to each primer for 1.15 min, and 72°C for 1.15 min; and a final cycle at 72°C for 7 min. The expected fragments for the primers and target genes [35,36] are listed in **Table 1**. Amplification products were visualized on a 2% agarose gel (Invitrogen®), stained with 5 mg/mL ethidium bromide, and visualized using a UV transilluminator (Major Science®; Saratoga, CA USA).

**Table 1:** *Salmonella* spp. research detection primers. Oligonucleotides used for *Salmonella* spp. confirmation and serovar detection of *Salmonella* spp.

Gene	Primer	Oligonucleotide sequence (5′→3′)	Size (bp)	Annealing temperature (°C)	Reference
*ompC*	*OMPCF* *OMPCR*	ATCGCTGACTTATGCAATCG CGGGTTGCGTTATAGGTCTG	204	57	[35]
*entF*	*ENTF* *ENTR*	TGTGTTTTATCTGATGCAAGAGG TGAACTACGTTCGTTCTTCTGG	304	56	[35]
*viaB*	*ViaBF* *ViaBR*	CACGCACCATCATTTCACCG AACAGGCTGTAGCGATTTAGG	738	57	[37]
DT 104	104F 104R	ATGCGTTTGGTCTCACAGCC GCTGAGGCCACGGATATTTA	102	56	[38]

### Antibiogram and assessment of antimicrobial resistance and virulence genes

The antibiogram test was performed on all identified microorganisms as described by Kirby-Bauer [39] with a disk diffusion assay, using Mueller–Hinton agar (Acumedia®). The antibiotics tested were amoxicillin (10 μg), ampicillin (10 μg), nalidixic acid (30 μg), colistin (10 μg), cefazolin (30 μg), ceftazidime (30 μg), ciprofloxacin (5 μg), chloramphenicol (30 μg), doxycycline (30 μg), streptomycin (10 μg), gentamicin (10 μg), tetracycline (30 μg), and sulfonamide (30 μg). The results were based on the Clinical and Laboratory Standards Institute [40] halo parameters, except for colistin standards, for which used the parameters defined by the European Committee on Antimicrobial Susceptibility Testing [41]. The presence of 17 antimicrobial resistance genes were investigated using the oligonucleotide sequences described in **Table 2**.

**Table 2:** Resistance genes. Oligonucleotides used for the antimicrobial resistance gene detection.

Antibiotic class	Gene	Primer	Nucleotide sequence (5′→3′)	Size (bp)	Annealing temperature (°C)	Reference
Polymyxins	*MCR- 1*	CLR5-F CLR5-R	CGGTCAGTCCGTTTGTTC CTTGGTCGGTCTGTAGGG	309	52	[42]
	*MCR- 2*	MCR2-F MCR2-R	TGGTACAGCCCCTTTATT GCTTGAGATTGGGTTATGA	1617	48	[43]
	*MCR- 3*	MCR3-F MCR3-R	TTGGCACTGTATTTTGCATTT TTAACGAAATTGGCTGGAACA	542	52	[44]
	*MCR- 4*	Mcr-4 FW Mcr-4 RV	ATTGGGATAGTCGCCTTTTT TTACAGCCAGAATCATTATCA	487	51	[45]
Tetracyclines	*tet*(A)	tet(A)-F tet(A)-R	GTGAAACCCAACATACCCC GAAGGCAAGCAGGATGTAG	887	53	[46]
	*tet*(B)	tet(B)-F tet(B)-R	CCTTATCATGCCAGTCTTGC ACTGCCGTTTTTTCGCC	773	53	[46]
	*tet*(C)	tet(C)-F tet(C)-R	ACTTGGAGCCACTATCGAC CTACAATCCATGCCAACCC	880	53	[46]
	*tet*(M)	tet(M)-1 tet(M)-2	GTTAAATAGTGTTCTTGGAG CTAAGATATGGCTCTAACAA	700	49	[47]
Macrolides	*ermA*	ermA-F ermA-R	TCTAAAAAGCATGTAAAAGAA CTTCGATAGTTTATTAATATTAGT	645	51	[48]
	*ermB*	ermB-F ermB-R	GAAAAGGTACTCAACCAAATA AGTAACGGTACTTAAATTGTTTAC	639	54	[48]
	*ermC*	ermC-F ermC-R	TCAAAACATAATATAGATAAA GCTAATATTGTTTAAATCGTCAAT	642	51	[48]
	*ereA*	ere(A)-F ere(A)-R	GCCGGTGCTCATGAACTTGAG CGACTCTATTCGATCAGAGGC	419	59	[46]
Amphenicols	*cat1*	CATIF CATIR	AGTTGCTCAATGTACCTATAACC TTGTAATTCATTAAGCATTCTGCC	547	58	[46]
	*cmlA*	cmlA-F cmlA-R	CCGCCACGGTGTTGTTGTTATC CACCTTGCCTGCCCATCATTAG	698	58	[46]
Sulfonamide	*sull*	sull-F sulI-R	TTCGGCATTCTGAATCTCAC ATGATCTAACCCTCGGTCTC	822	53	[46]
β-lactams	*blaSHV*	blaSHV-F blaSHV-R	TCGCCTGTGTATTATCTCCC CGCAGATAAATCACCACAATG	768	51	[46]
	*ampC*	AmpC-For AmpC-Rev	TTCTATCAAMACTGGCARCC CCYTTTTATGTACCCAYGA	550	49	[49]
Aminoglycosides	aac(3)-I	aac(3)-I-F aac(3)-I-R	ACCTACTCCCAACATCAGCC ATATAGATCTCACTACGCGC	157	54	[46]

For research on virulence genes, 12 virulence markers were selected based on their ability to cause lesions in the host organism [50,51]. The oligonucleotide annealing temperatures are listed in **Table 3**.

**Table 3:** Virulence genes and *E*. *coli* serotypes. Oligonucleotides used for virulence gene and serotype detection in *E*. *coli* strains.

Gene	*Primer*	Oligonucleotides sequence (5′→3′)	Size (bp)	Annealing temperature (°C)	References
*Tir α*	B139 B152	CAGCCTGCCACTTACCTTCACA CGCTAACCTCCAAACCATT	781	54.2	[52]
*Tir β*	B139 B140	CAGCCTGCCACTTACCTTCACA TGTATGTCGCACTCTGATT	342	53.4	[52]
*Tir γ*	*B139* *B141*	CAGCCTGCCACTTACCTTCACA GTCGGCAGTTTCAGTTTCAC	560	54.7	[52]
*Stx1*	stx1F stx1R	AGAGCGATGTTACGGTTTG TTGCCCCCAGAGTGGATG	388	50	[53]
*Stx2*	stx2F stx2R	TGGGTTTTTCTTCGGTATC GACATTCTGGTTGACTCTCTT	807	45	[53]
*Eae*	eaeAF eaeAR	AGGCTTCGTCACAGTTG CCATCGTCACCAGAGGA	570	48	[53]
*hlyA*	hlyAF hlyAR	GCATCATCAAGCGTACGTTCC AATGAGCCAAGCTGGTTAAGCT	534	57	[54]
*Saa*	SAADF SAADR	CGTGATGAACAGGCTATTGC ATGGACATGCCTGTGGCAAC	119	55	[55]
*EspP*	*esp-A* *esp-B*	AAACAGCAGGCACTTGAACG GGAGTCGTCAGTCAGTAGAT	1830	56	[56]
*O111*	O111F O111R	TAGAGAAATTATCAAGTTAGTTCC ATAGTTATGAACATCTTGTTTAGC	406	60	[54]
*O113*	O113F O113R	AGCGTTTCTGACATATGGAGTG GTGTTAGTATCAAAAGAGGCTCC	593	60	[56]
*O157*	O157F O157R	CGGACATCCATGTGATATGRG TTGCCTATGTACAGCTAATCC	259	60	[54]

Amplification reactions were performed in a final volume of 25 μL, containing 2 U Taq DNA polymerase (Invitrogen®), 2 mM phosphated deoxyribonucleotides (Invitrogen®), 1× buffer (200 mM Tris-HCl, pH 8.4, 500 mM KCl; Invitrogen®), 3mM MgCl_2_ (Invitrogen®), and 1 μM primers. The reaction was performed in a MyCycler thermal cycler (BioRad®) under the following conditions: initial denaturation at 94°C for 3 min; 30 cycles at 94°C for 30 sec, annealing temperature according to each primer for 30 sec, and 72°C for 30 sec, and a final cycle at 72°C for 10 min. Amplification products were visualized on a 2% agarose gel (Invitrogen®), stained with 5 mg/mL ethidium bromide, and visualized using a transilluminator (Major Science®).

### Evaluation of *in vitro* biofilm-forming capability

The *in vitro* biofilm-forming capability was evaluated as described by Agostinho Davanzo *et al*. [57] and Borges *et al*. [15].

The 96-well polystyrene titration microplates (Kartell®; Noviglio, Italy) containing the strains were incubated for 24 and 72 h at 37°C (near optimal temperature for target microorganism multiplication [58]), 24°C (average ideal temperature for extracellular polymeric matrix component expression [59,60]), and 10°C (maximum temperature recommended by the Brazilian Federal Inspection Service for facilities intended for the roasting and deboning of carcasses from cooling [61]).

The mean absorbance obtained from triplicate readings was used to determine the final optical density of each strain (ODf), which was compared with that of the negative control (ODn). The isolates were categorized into non-biofilm-forming isolates (NF) when ODf ≤ ODn, weakly biofilm-forming when ODn < ODf ≤ 2× ODn, moderate biofilm-forming when 2× ODn < ODf ≤ 4× ODn, or strong biofilm-forming when 4× ODn < ODf [62].

Statistical analyses were performed using SAS software (v9.4; Cary, NC, USA) at 5% significance level. Initially, a normality test was performed (Shapiro–Wilk), and the data were subjected to analysis of variance using PROC GLIMMIX. The variables included time, detection points, temperature, and their interactions.

This study did not require permission from an ethics committee as no human or animal experimentation was involved.

## Results

### *E*. *coli* detection and isolation

Twenty-one (47.72%) *E*. *coli* strains were detected in the swabs collected from the environment, utensils, and equipment of swine slaughterhouses, with 9 and 12 isolates being obtained from slaughterhouses A and B, respectively, between 2019 and 2021. The detection points for each *E*. *coli* isolate, as well as the total number of isolates per collection point, are listed in **Table 4**.

**Table 4:** *E*. *coli* detection points. Points of *E*. *coli* detection in the environment, equipment, and utensils of swine slaughterhouses A and B located in the Federal District of Brazil.

*E*. *coli* detection points in swine slaughterhouses	Visit 1 Slaughterhouse A	Visit 2 Slaughterhouse A	Visit 1 Slaughterhouse B	Visit 2 Slaughterhouse B	Total *E*. *coli* isolates
**Chute of viscera**	1	0	0	0	1
**Drains (dirty area)**	1	0	1	0	2
**Bleeding knife**	0	1	1	0	2
**Drains (clean area)**	0	1	1	0	2
**Viscera table**	1	0	1	0	2
**Dehairing machine**	1	1	0	0	2
**Table**	0	0	1	1	2
**Carcass splitting saw**	0	0	1	1	2
**Clean area wall**	0	0	1	0	1
**Floor cooling chamber**	1	0	1	1	3
**Walls cooling chamber**	0	1	1	0	2
**Total swabs**	5	4	9	3	21

*E*. *coli* was most commonly detected from the swabs of the carcass cooling chamber floor (75% samplings), and least commonly from the swabs of the viscera kicker and the wall present in the clean area of the slaughter room (25% for both locations). *E*. *coli* was not detected in the toilet table, carcass splitting saw, or the clean area wall of the slaughter room of slaughterhouse A, as well as the viscera kicker and dehairing machine of slaughterhouse B during either visit.

### *Salmonella* spp. detection and isolation

Only one (2.27%) isolate of *Salmonella* spp. was detected from the 44 swab samples collected from swine slaughterhouses A and B. The isolate was recovered in the carcass cooling chamber during the second visit to slaughterhouse A.

**Fig 1:**
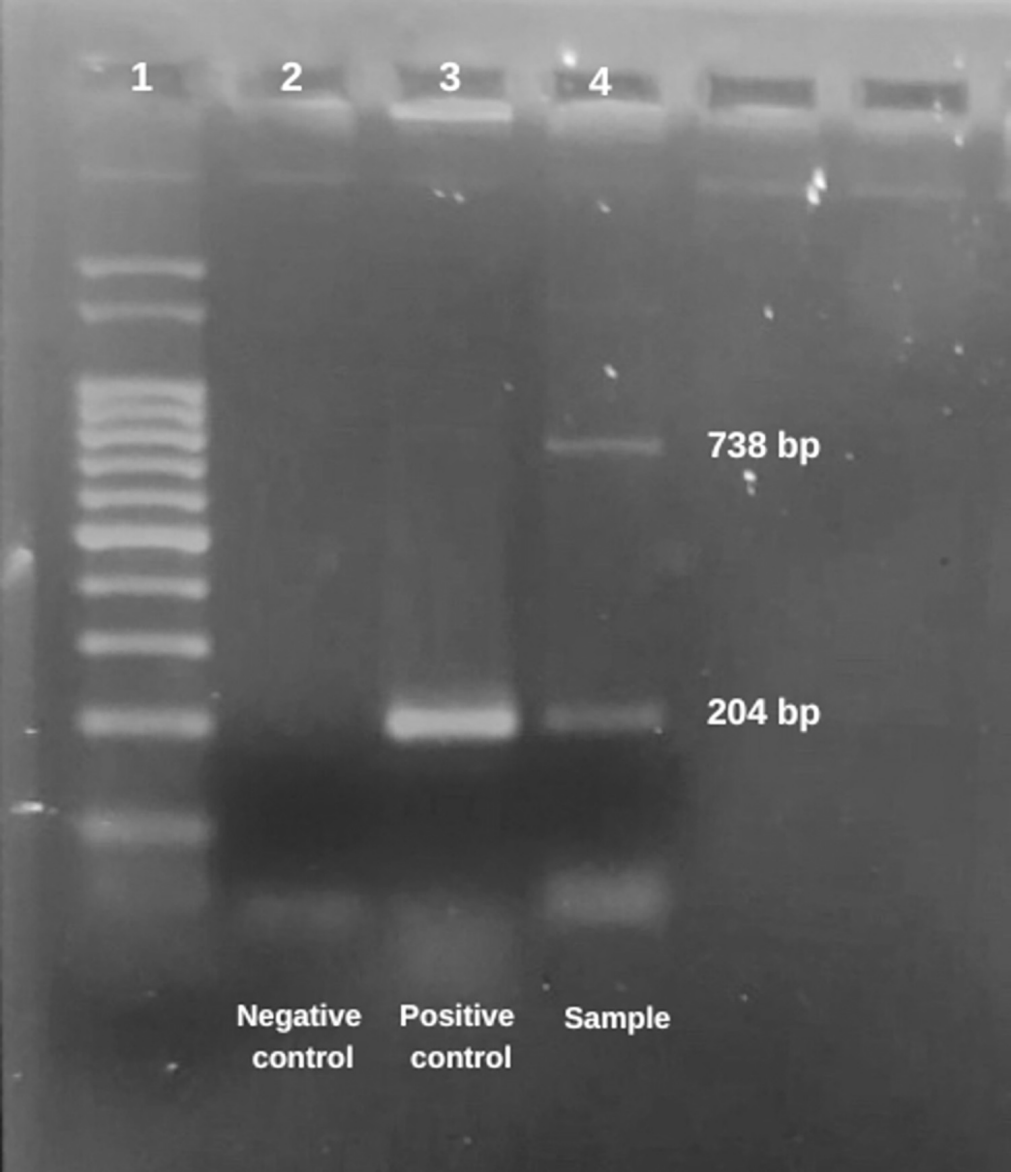
*Salmonella* Typhi. PCR confirmation of *S*. Typhi isolated from slaughterhouse A located in the Federal District of Brazil. 1) 100-bp marker (Invitrogen®), 2) negative control, 3) positive control for *Salmonella* spp., 204-bp fragment (*ompC* primer), 4) 204-bp fragment (*ompC* primer) for *Salmonella* spp. and 738-bp fragment (*viaB* primer) for Typhi serotype. Visualization on a 2% agarose gel stained with 0.5 μg/mL ethidium bromide in an ultraviolet transilluminator (Major Science®).

The *Salmonella* genus was confirmed (204-bp fragment) and the *S*. Typhi serotype (738-bp fragment) was identified by colony PCR (**Fig 1**).

### *L*. *monocytogenes* detection

*L*. *monocytogenes* was not detected in any of the swab samples in the present study.

### Antibiogram and resistance genes of *E*. *coli* isolates

All 21 *E*. *coli* isolates were resistant or showed intermediate sensitivity to 12 of the 13 antimicrobials tested; 20 (95.2%) isolates were resistant to ampicillin and chloramphenicol each, 18 (85.8%) to amoxicillin, 17 (80.95%) to streptomycin and tetracycline each, 13 (61.9%) to sulfonamide, 12 (57.15%) to nalidixic acid and doxycycline each, 11 (52.4%) to cefazolin, seven (33.3%) to ciprofloxacin, five (23.8%) to gentamicin, and two (9.52%) to colistin. Moreover, six (28.6%) isolates presented intermediate resistance to ciprofloxacin, four (19.05%) to streptomycin, two (9.52%) to nalidixic acid, and one (4.8%) to chloramphenicol, cefazolin, and gentamicin each. None of the 21 isolates tested was resistant to ceftazidime (**Table 5)**.

**Table 5:** *E*. *coli* antibiograms. Antibiogram results of 21 *E*. *coli* isolates from swine slaughterhouses A and B.

Antibiotic class	Antimicrobial	Number of resistant isolates (%)	Number of intermediate resistance isolates (%)	Number of sensitive isolates (%)	Total resistant and intermediate isolates (%)
Polymyxins	Colistin (COL)	2 (9.52%)	0 (0%)	19 (90.48%)	2 (9.52%)
Tetracyclines	Tetracycline (TET)	17 (80.95%)	0 (0%)	4 (19.05%)	17 (80.95%)
	Doxycycline (DOX)	12 (57.15%)	2 (9.52%)	7 (33.33%)	14 (66.7%)
Amphenicols	Chloramphenicol (CLO)	20 (95.2%)	1 (4.8%)	0 (0%)	21 (100%)
Sulfonamides	Sulfonamide (SUL)	13 (61.9%)	0 (0%)	8 (38.1%)	13 (61.9%)
β-lactams	Amoxicillin (AMO)	18 (85.8%)	0 (0%)	3 (14.2%)	18 (85.8%)
	Ampicillin (AMP)	20 (95.2%)	0 (0%)	1 (4.8%)	20 (100%)
Aminoglycosides	Streptomycin (EST)	17 (80.95%)	4 (19.05%)	0 (0%)	21 (100%)
	Gentamicin (GEN)	5 (23.8%)	1 (4.8%)	15 (71.4%)	6 (28.6%)
Cephalosporins	Cefazolin (CFZ)	11 (52.4%)	1 (4.8%)	9 (42.8%)	12 (57.15%)
	Ceftazidime (CAZ	0 (0%)	0 (0%)	21 (100%)	0 (0%)
Fluoroquinolones	Ciprofloxacin (CIP)	7 (33.3%)	6 (28,6%)	8 (38,1%)	13 (61.9%)
	Nalidixic acid (NAL)	12 (57.15%)	2 (9.52%)	7 (33.33%)	14 (66.7%)

Twelve isolates expressed a resistance phenotype, and the antibiogram results were confirmed by a resistance gene detection (**Table 6**). Isolates 1 and 6 were resistant to ampicillin and tetracycline in the antibiogram and possess the respective genes *ampC* and *tet(A)*; isolates 10 and 17 were resistant to tetracycline in the antibiogram and possess the *tet(A)* gene; isolates 5 and 32 were resistant to chloramphenicol in the antibiogram and possess the genes *clmA* and *cat1*; isolate 14 was resistant to tetracycline and sulfonamide in antibiogram and possess *tet(A)*, *tet(B)*, and *sulI* genes; isolate 15 was resistant to tetracycline and colistin and possess *MCR-1*, *MCR-3*, and *tet(B)* genes; isolate 32 was resistant to chloramphenicol and possess the *cat1* gene; isolate 33 was resistant to ampicillin and possess the genes *ampC* and *blaSHV*; isolate 41 was resistant to ampicillin and tetracycline and possess the genes *ampC* and *tet(B);* and isolate 43 was also resistant to ampicillin and possess the gene *ampC*. The isolates 1, 10, and 40 possess the resistance genes *sulI*, *MCR-3*, and *tet(B)*, respectively, but were sensitive to sulfonamide and colistin in the antibiogram. In this study, we could not relate the *aac(3)-I* to the aminoglycosides and *ermA*, *ermB*, *ermC*, and *ereA* to the macrolides.

**Table 6:** *E*. *coli* genes’ detection and antibiograms. Results of 21 E. coli antibiograms, detection of resistance and virulence genes, and detection points in slaughterhouses A and B.

*E*. *coli* isolate	Swine slaughterhouse	Detection point	Antibiogram Resistant	Antibiogram Intermediate resistance	Antibiogram Sensitive	Resistance genes	Virulence genes
**1**	A	Chute of visceras	NAL, AMO, AMP, CFZ, CLO, EST, TET	–	CAZ, CIP, COL, DOX, GEN, SUL	*ampC*, *tet(A)*, *sulI*	*hlyA*, *tir β*, *stx-1*
**2**	A	Drains (clean area)	NAL, AMO, AMP, CLO, DOX, EST, TET, SUL	CFZ	CAZ, CIP, COL, GEN	–	*eae*, *hlyA*, *tir α*, tir β
**5**	A	Evisceration table	AMO, AMP, CFZ, CLO, DOX, TET, SUL	EST, NAL	CAZ, CIP, COL, GEN	*clmA*	*Stx-1*, *tir-β*
**6**	A	Dehairing machine	NAL, AMO, AMP, CFZ, CLO, DOX, TET, SUL	CIP, EST	CAZ, COL, GEN	*ampC*, *tet(A)*	*tir α*, *tir γ*
**10**	A	Floor (cooling chamber)	NAL, AMO, AMP, CFZ, CIP, CLO DOX, EST, GEN, TET, SUL	–	CAZ, COL	*MCR-3*, *tet(A)*	*tir α*
**14**	A	Bleeding knife	NAL, AMO, AMP, CIP, CLO, DOX, EST, TET, SUL	–	CFZ, CAZ, COL, GEN	*tet(A)*, *tet(B)*, *sulI*	–
**15**	A	Drains (clean area)	NAL, AMO, AMP, CFZ, CIP, CLO, COL, DOX, EST, GEN, TET, SUL	–	CAZ	*MCR-1*, *MCR-3*, *tet(B)*	*tir α*
**17**	A	Dehairing machine	NAL, AMO, AMP, CFZ, CIP, CLO, DOX, EST, GEN, TET, SUL	–	CAZ, COL	*tet(A)*	*Saa*
**22**	A	Walls (cooling chamber)	AMO, AMP, CFZ, DOX, EST, TET	NAL, CIP, CLO	CAZ, COL, GEN, SUL	–	*tir α*, *tir β*, *tir γ*
**24**	B	Drains (dirty area)	AMO, AMP, CFZ, CIP, CLO, EST, TET, SUL	DOX	NAL, CAZ, COL, GEN	–	–
**25**	B	Bleeding knife	CLO, EST, TET	DOX	NAL, AMO, AMP, CAZ, CFZ, CIP, COL, GEN, SUL	–	*stx-1*
**26**	B	Drains (clean area)	NAL, AMO, AMP, CIP, CLO, DOX, EST, GEN, TET, SUL	–	CFZ, CAZ, COL	–	–
**27**	B	Evisceration table	NAL, AMO, AMP, CLO, COL, EST, TET, SUL	–	CFZ, CAZ, CIP, DOX, GEN	–	–
**29**	B	Table (dirty area)	AMO, AMP, CLO, EST, TET, SUL	–	NAL, CFZ, CAZ, CIP, COL, DOX, GEN	–	–
**30**	B	Carcass splitting saw	AMO, AMP, CLO, EST, TET	CIP	NAL, CFZ, CAZ, COL, DOX, GEN, SUL	–	–
**31**	B	Wall (dirty area)	NAL, AMO, AMP, CLO, DOX	CIP, EST	CFZ, CAZ, COL, GEN, TET, SUL	–	*Stx-1*
**32**	B	Floor (cooling chamber)	AMP, CFZ, CLO, EST	–	NAL, AMO, CAZ, CIP, COL, DOX, GEN, TET, SUL	*cat1*	*Stx-1*
**33**	B	Wall (cooling chamber)	AMP, CFZ, CLO	EST	NAL, AMO, CAZ, CIP, COL, DOX, GEN, TET, SUL	*ampC*, *blaSHV*	–
**40**	B	Table (dirty area)	NAL, AMO, AMP, CFZ, CLO, EST	CIP, GEN	CAZ, COL, DOX, TET, SUL	*tet(B)*	*tir γ*
**41**	B	Carcass splitting saw	NAL, AMO, AMP, CIP, CLO, DOX, EST, GEN, TET, SUL	–	CFZ, CAZ, COL	*ampC*, *tet(B)*	*tir β*
**43**	B	Floor (cooling chamber)	AMO, AMP, CLO, DOX, EST, TET	CIP	NAL, CFZ, CAZ, COL, GEN, SUL	*ampC*	–

* The inhibition zone diameters were measured and interpreted according to the CLSI [40] parameters, except for the standards for colistin, in which the parameters were defined by EUCAST [41].

** In isolates without resistance/virulence genes, consider only the ones detected by the primers used in this study.

### Antibiogram and resistance genes of *Salmonella* spp.

The sole *S*. Typhi isolate was resistant to 8 of the 13 antimicrobials tested, including nalidixic acid, cefazolin, chloramphenicol, doxycycline, streptomycin, gentamicin, tetracycline, and sulfonamide. The isolate was sensitive to amoxicillin, ampicillin, ciprofloxacin, ceftazidime, and colistin. Intermediate resistance to any of the investigated antimicrobials was not detected. Moreover, the antimicrobial resistance gene, *ampC*, which corresponds to ß-lactams, as well as *tet(B)*, *tet(C)*, and *tet(M)*, which corresponds to tetracyclines, were detected. The resistance to tetracycline and doxycycline were confirmed by the presence of *tet(B)*, *tet(C)*, and *tet(M)*. The *Salmonella* spp. isolate was sensitive to ampicillin, an antibiotic of ß-lactam class, and possesses the *ampC* gene, indicating ß-lactam resistance (**Table 7).**

**Table 7:** *Salmonella* spp. results of the antibiogram. Results of the *Salmonella* spp. isolate antibiogram by disk diffusion, antimicrobial resistance gene detection of *Salmonella* spp. isolate, and point of isolation point at swine slaughterhouse A located in the Federal District of Brazil.

*Salmonella* Typhi	Swine slaughterhouse identification	Detection point	Antibiogram resistance	Antibiogram sensitivity	Resistance genes detected
21	A	Floor (cooling chamber)	CFZ		
			NAL	AMP	*ampC*
			CLO	AMO	–
			SUL	CIP	–
			EST	CAZ	–
			GEN	COL	–
			DOX	–	–
			TET	–	*tet(B)*, *tet(C)*, *tet(M)*

Additionally, *S*. Typhi presented an antimicrobial-resistant phenotype to cefazolin, nalidixic acid, chloramphenicol, sulfonamide, streptomycin, gentamicin, tetracycline, and doxycycline. However, no resistant genes were investigated in this study against the drugs, such as *cat1* and *clmA* for chloramphenicol (amphenicols), *aac(3)-I* for streptomycin and gentamicin (aminoglycosides), or *sul1* for sulfonamides.

In addition, no resistance genes for polymyxins (*MCR-1*, *MCR-2*, *MCR-3*, and *MCR-4*) or macrolides (*ermA*, *ermB*, *ermC*, and *ereA*) were detected.

### Virulence genes in *E*. *coli* isolates

Seven of the nine investigated virulence genes were detected in the *E*. *coli* isolates. Thirteen (61.9%) of the 21 *E*. *coli* isolates presented at least one virulence gene, of which five (23.8%), isolates harbored *tir α*, five (23.8%) harbored *tir β*, five (23.8%) harbored *stx-1*, three (14.2%) harbored *tir γ*, three (14.2%) harbored *hlyA*, one (4.8%) harbored *eae*, and one (4.8%) presented *saa*. Virulence genes *stx-2* and *Esp* were not detected.

As for the investigated serotypes, two (9.5%) isolates presented serotype *O157* (isolates 15 and 22). *O111* and *O113* serotypes were not detected in this study. The individual isolate results, detection point in the industry, antibiogram results, and detection results of antimicrobial resistance genes are presented in **Table 3**.

### Evaluation of *in vitro* biofilm formation capacity of *E*. *coli* isolates

Biofilm-forming capacity increased after incubating for 72 h, and the optical density at 24 h indicated an initial stage of adherence. Biofilm-forming capacity was the highest after incubating at 10°C, while it was the lowest after incubating at 37°C for 24 h. After incubating for 72 h at 37°C, 2 (9.5%), 6 (28.6%), 11 (52.4%), and 2 (9.5%) *E*. *coli* isolates showed strong, moderate, weak, and no biofilm-forming capacity, respectively. Interestingly, 4 (19.05%), 10 (47.75%), 6 (28.6%), and 1 (4.8%) isolates showed strong, moderate, weak, and no biofilm-forming capacity, respectively, at 24°C; furthermore, 4 (19.05%), 8 (38.1%), 7 (33.3%), and 2 (9.5%) isolates showed strong, moderate, weak, and no biofilm-forming capacity, respectively, at 10°C. According to the statistical analyses performed, biofilm formation capacity was significantly different at the 5% significance level (*P* < 0.0001) in relation to different temperatures, incubation periods, and swab detection points.

Individual identification, as well as the optical density and classification of biofilm-forming capacity of the 21 *E*. *coli* isolates after incubating at the three temperatures for 24 and 72 h are presented in **S1 Table**. Concerning the detection points, isolate 40 had the highest biofilm-forming capacity, while isolate 22 (isolated from the carcass cooling chamber wall) did not form biofilms at any time or temperature conditions.

### Evaluation of *in vitro* biofilm formation capacity of *Salmonella* spp. isolates

*S*. Typhi incubated for 24 h at 37, 24, and 10°C showed weak biofilm-forming capacity at 37 and 24°C and did not form biofilms at 10°C. Moderate biofilm formation was observed when incubated for 72 h at 37°C, and weak biofilm formation was observed at 24 and 10°C. The optical densities as well as the biofilm-forming capacities of *S*. Typhi are described in **Table 8**.

**Table 8:** *Salmonella* Typhi biofilm formation. *In vitro* biofilm-forming capacity of *S*. Typhi after 24 and 72 h incubation at 37, 24, and 10°C.

*Salmonella* isolate	Incubation period	ODf at 37°C	ODf at 24°C	ODf at 10°C	Classification at 37°C	Classification at 24°C	Classification at 10°C
**21**	24h	0.089	0.112	0.084	weak	weak	NF
	72h	0.135	0.074	0.084	moderate	weak	weak

* The classification is based on the parameters described by Stepanović *et al*. [62], where ODf is the final optical density of the isolates, and ODn is the negative control optical density. ODn = 0.064 and 0.086 in isolates incubated for 24 and 72 h, respectively. The isolates were classified into non-biofilm-forming (NF, ODf ≤ ODn), weak biofilm-forming (ODn < ODf ≤ 2× ODn), moderate biofilm-forming (2× ODn < ODf ≤ 4× ODn), or strong biofilm-forming (4× ODn < ODf) according to their biofilm-forming ability and intensity.

## Discussion

### *E*. *coli* isolation from swine slaughterhouses located in the Federal District of Brazil

The microorganisms detected from the dehairing machine, tables, carcass splitting saw, and carcass cooling chamber floor swabs collected during both visits at slaughterhouses A and B suggest *E*. *coli* permanence and distribution in the slaughter process, corroborating the presence of *E*. *coli* on floors, tables, and knives of swine slaughterhouses in Nigeria [63]. In addition, Namvar & Warriner [64] detected the permanence of *E*. *coli* on swine slaughterhouse floors in two swabs collected on different dates. In general, the presence of *E*. *coli* in the swine slaughterhouse environment may indicate cleaning process failure [65]. Moreover, repeated isolation from the same industrial collection points may suggest the presence of bacterial biofilms [64]. Even a one-time *E*. *coli* recovery may indicate cross-contamination [66]. The presence of *E*. *coli* in dehairing machines in this study may be due to the presence of microorganisms in pig bristles, which are directed to the dehairing machines after slaughter and can contaminate the water and blades of the equipment [65]. A failure in equipment sanitization procedures may also cause bacterial contamination.

### *Salmonella* spp. isolation in swine slaughterhouses located in the Federal District of Brazil

*Salmonella* spp. have also been detected in the environment, equipment, and utensils of swine slaughterhouses in other European countries, such as Italy [67], Belgium [68], and the Netherlands [69], with frequent contamination points being the carcass splitting saw and knives used. The presence of *Salmonella* spp. in the carcass cooling chamber may be related to cross-contamination during the slaughter process, swine carcass cooling, and failures related to hygiene procedures. Botteldoorn *et al*. [68] have discussed the difficulty in stating the origin of the contamination site, since it may vary depending on the number of animals slaughtered daily in the establishment, pig farming practices responsible for raising and breeding domestic pigs as livestock for slaughter, failures in the conduction of standard sanitation operating procedures, and even failures in employee training. *S*. Typhi detection in the Brazilian slaughterhouses in this study is relevant because of the possibility of carcass cross-contamination when stored in the cooling chamber. This goes against Normative Instruction 79 [70], which fosters the importance of this specific microbiological analysis when approving risk-based ante- and post-mortem pig inspection procedures.

### Studies on *L*. *monocytogenes* in swine slaughterhouses located in the Federal District of Brazil

The non-isolation of *L*. *monocytogenes* from a swine slaughter facility in this study diverges from that reported by Moreno *et al*. [71] and Sereno *et al*. [72] in Brazil, Lariviere-Gauthier *et al*. [73] in Canada, Autio *et al*. [74] in Finland, and Morganti *et al*. [75] in Italy. A possible hypothesis for the non-detection of this microorganism would be the correct performance of the standard sanitation operating procedures in slaughterhouses, which may have been favored by the average size of the participating industries in this study, which slaughtered 110 animals per day, allowing better control of daily hygiene procedures. The non-detection of *L*. *monocytogenes* may also have occurred because of the restricted number of samples collected due to the resistance of the local industries participating in this study. However, it is important to emphasize that the non-detection of *L*. *monocytogenes* does not ensure its absence in slaughterhouses in the Federal District of Brazil since the microorganism presents a cosmopolitan characteristic [76,77]. Moreover, it has been detected in bovine meat cuts and the environment of bovine slaughterhouses in the Federal District [78]. In addition, this microorganism has also been detected in minced beef and hot dog sausages commercialized in this region [79].

### Antibiogram and antimicrobial resistance gene detection in *E*. *coli* isolates

The existence of multidrug-resistant isolates, such as isolates 10, 15, and 17, which are resistant to nalidixic acid, amoxicillin, ampicillin, cefazolin, ciprofloxacin, chloramphenicol, doxycycline streptomycin, gentamicin, tetracycline, and sulfonamide, is a public health concern as they may suggest the indiscriminate use of antibiotics for treatment, disease prevention, and growth promotion [80]. This may cause the emergence of resistant bacteria in livestock animals, their spread in the environment, or residues in animal products consumed by the population [80,81].

In this study, 95.2% (20/21) *E*. *coli* isolates were chloramphenicol-resistant, which is important because it is a broad-spectrum antibiotic with prohibited use in Brazil since 2003 according to Normative Instruction 09 [82]. The detection of resistant strains can be explained by the maintenance of resistance genes through co-selection with other resistance and virulence genes, often linked to transmissible/mobile genetic elements [83]. However, further studies should be conducted to verify the possible origins of this antimicrobial resistance because only two *E*. *coli* isolates, 5 and 32, with *cat1* and *clmA* genes respectively, were chloramphenicol-resistant.

Chloramphenicol resistance has also been reported years after the ban (in the 1980s) on its use in animal feed in the USA. Chloramphenicol resistance was detected in 53% *E*. *coli* strains from diarrheic pigs, along with *clmA* [84]. This persistence is explained by the location of the *cmlA* in the class 1 integrins, allowing transfer by conjugation as they are linked to other genes encoding resistance to antimicrobials currently allowed for use in animal feed [84], which may also explain the resistance found in this study. In Japan, Harada *et al*. [85] corroborated this information by showing that *clmA* and *cat1* are involved in co-resistance, contributing to chloramphenicol-resistant strain selection, allowing it to persist despite its ban in swine feed, which may also explain the presence of resistance found in this study.

Isolate 15 was colistin resistant and possessed the resistance genes *MCR-1* and *MCR-3*, indicating polymyxin resistance. The World Health Organization (WHO) has classified polymyxins as critically important and the highest-priority antimicrobials [86]; thus, this result is concerning for public health. Other studies have shown that the use of this antibiotic increased in serious infection treatment in humans, and the presence of *MCR* genes confer transmissible resistance and spread resistant microorganisms through the food chain [80,87,88].

Some *E*. *coli* isolates showed antibiogram sensitivity and possessed resistance genes to the same antibiotic; isolate 1 harbored *sul1* and sensitivity to sulfonamide; isolate 10 was sensitive to colistin and contained *MCR-3*, whereas isolate 40 was sensitive to tetracycline and doxycycline, and *tet(B)* was detected, which can be explained by the non-expression of genes present in its bacterial or plasmid DNA [89].

### Antibiogram and antimicrobial resistance gene detection in *Salmonella* spp. isolates

As detected in the *E*. *coli* isolates, the resistance of *Salmonella* spp. to chloramphenicol is relevant to public health, since it has been banned from use in animal feed since 2003 [82]. This is problematic since food can be contaminated by resistant pathogens and distributed over large geographical areas, increasing antimicrobial resistance in the population that consumes such products [90]. Wu *et al*. [91] have also reported that *Salmonella* isolates from the environment and carcasses of pig slaughterhouses in China were chloramphenicol-resistant. Botteldoorn *et al*. [92] also detected a chloramphenicol-resistant microorganism in Belgian pig slaughterhouse environments, utensils, and carcasses.

As described previously, ampicillin (ß-lactam) sensitivity and *ampC* (ß-lactam) detection can be explained by the lack of expression of the gene present in its bacterial or plasmid DNA [93]. Consequently, findings such as phenotypic resistance to an antibiotic (amphenicols, aminoglycosides, and sulfonamides), and non-detection of correlated resistant genes, cannot be interpreted as the absence of resistance genes. The presence of cross-resistance [94,95] is considered valid in this case. Another possibility is the inappropriate methodology of primer choice, since classic primers were used in gene detection, and others such as *sul2*, *sul3*, and *floR* [96], were not included in this study. Furthermore, Schwan *et al*. [97] and Jeamsripong *et al*. [98] showed a concordance of phenotypic and genotypic AMR results of *Salmonella* spp. that represented the results different from those of this study. Therefore, to elucidate the origin of phenotype resistance, complete genome sequencing would be required.

### Virulence genes in *E*. *coli* isolates

*E*. *coli* isolate 2 possessed the highest number of virulence genes, including *hlyA*, *eae*, *tir α*, and *tir β*, which are associated with the antibiogram profile of resistance to nalidixic acid, amoxicillin, ampicillin, chloramphenicol, doxycycline, streptomycin, tetracycline, and sulfonamide. These findings imply the importance of *E*. *coli* isolate 2 due to the public health risks caused by the presence of these genes. *hlyA* encodes alpha-hemolysin exotoxin and is related to clinical infections in humans, such as pyelonephritis and sepsis [99]. *eae* and *tir* can be related to enteropathogenic *E*. *coli* strains, since *eae* encodes the adhesion factor intimin and *tir* is an intimin receptor, allowing the attaching and effacing pathogenesis mechanism, causing lesions in the intestinal mucosa of humans and animals [100,101]. The presence of these virulence genes in addition to the resistance to the antimicrobials mentioned suggests a potential risk to the population. Moreover, *E*. *coli* isolate 17 presented *saa* association with *tet(A)* and antibiogram resistance profile to nalidixic acid, amoxicillin, ampicillin, cefazolin, ciprofloxacin, chloramphenicol, doxycycline, streptomycin, gentamicin, tetracycline, and sulfonamide. It implies a risk to public health since the isolate is resistant to multiple antibiotics and possesses *saa*, which may lead to clinical cases of severe diarrhea in humans [102].

Additionally, it is important to highlight that 5/21 *E*. *coli* isolates detected in post-sanitation locations of processing plants were non-O157:H7 Shiga toxin-producing *E*. *coli* strains (STECs). The presence of *stx-1* in non-*O157* strains, as observed in isolates 1, 5, 25, 31, and 32, even though they are not from a serotype conventionally associated with pathologies (*O157*:*H7*), is associated with severe disease in humans [103,104]. The *E*. *coli* virulence genes detected in this study confer pathogenicity and are a potential risk to public health [105]; the isolates were isolated from the surfaces of equipment, utensils, and the environment of swine slaughterhouses, having direct and/or indirect contact with the food produced. This may cause direct contamination or cross-contamination of final products that will be consumed by the population of the Federal District area and other Brazilian states.

The virulence genes present in isolates from slaughterhouses A and B were different, which can be attributed to the different batches of animals received for slaughter and different sanitary management in livestock animal farms [106]. Although very few studies have verified virulence genes in *E*. *coli* detected in the environment, equipment, and utensils in swine slaughterhouses/carcasses, some studies have detected these virulence genes in pig carcasses [107–110], suggesting that the virulence genes investigated in this study are circulating in *E*. *coli* strains in pigs. In addition to the potential public health risk related to food contamination, it is important to emphasize the economic loss due to infection by pathogenic *E*. *coli* strains in pigs (particularly piglets) and feed conversion reduction due to diarrheal symptoms, which may contaminate other pigs on the farm and cause death due to severe dehydration or the development of syndromes related to pathogenic *E*. *coli* strains [111].

### *In vitro* evaluation of biofilm-forming capacity of *E*. *coli* isolates

The biofilm formation in most *E*. *coli* isolates was the maximum after incubating for 72 h at 10°C. This corroborates with the guidelines of the Brazilian Ministry of Agriculture, Livestock and Supply Ordinance No. 1304 [112] about the importance of daily cleaning in slaughterhouses after the activities and before starting the slaughter process, aiming for hygienic sanitary quality of produced food. It is relevant that the highest capacity to form biofilms occurred at refrigeration temperature (10°C); this condition resembles that of the climate-controlled deboning in Brazilian slaughterhouses [112]. Therefore, *E*. *coli* strains forming biofilms at this temperature can represent a contamination risk of the final food product.

Five (23.8%) *E*. *coli* isolates showed strong biofilm-forming capacities in at least one of the three temperatures tested, among which, isolates 32 and 40 harbored *cat1* and *tet(B)*, respectively. Moreover, 15 (71.4%) isolates showed moderate biofilm-forming capacity at all temperatures tested and antimicrobial resistance genes were detected in nine of the 15 isolates: isolate 1 (*ampC* and *tet(A)*, and *sul1*), isolate 5 (*clmA*), isolate 6 (*tet(A)* and *ampC*), isolate 10 (*tet(A)* and *MCR-3*), isolate 17 (*tet(A)*), isolate 33 (*ampC* and *blaSHV*), isolate 40 (*tet(B)*), isolate 41 (*tet(B)* and *ampC*), and isolate 43 (*ampC*). These results show the importance of *E*. *coli* isolates due to the risk posed to public health and the capacity to spread antimicrobial resistance in the environment [113]. In addition to the presence of resistance genes, these isolates harbored virulence genes *stx-1*, *saa*, *hlyA*, and *tir*, which are associated with serious disease development in humans, reinforcing the potential risk to consumers of the meat processed in meatpacking industries.

Bacterial biofilm formation is a serious problem in food industries [114] as it allows microorganisms to remain viable for months on surfaces after sanitization and hygiene procedures, becoming a recurrent point of contamination [115]. The recurring failure of sanitization processes causes bacterial attachment to abiotic surfaces; once established in the environment, removing the biofilm is challenging in food industries as a self-produced extracellular matrix enables the adhesion of other microorganisms and the colonization of several surfaces [116]. In industrial environments, complex multi-species communities permit bacterial cell attachment and detachment, enabling product cross-contamination and, in turn, product shelf-life reduction and disease transmission [117]. This is the first study to evaluate the biofilm formation capacity of *E*. *coli* in swine slaughterhouses in Brazil.

### *In vitro* evaluation of *Salmonella* spp. biofilm-forming capacity

*S*. Typhi presented a moderate biofilm-forming capacity at 37°C after incubation for 72 h, which may have occurred because of its ideal growth temperature [118]. Moreover, the isolate showed weak biofilm-forming capacity or did not form biofilms at other temperatures and incubation periods even though it was detected in the environment. Very few studies have evaluated the biofilm-forming capacity of *Salmonella* spp. in Brazilian poultry slaughterhouses; the results of this study were similar to those reported by Garcia *et al*. [119], which reported weak and moderate biofilm-forming capacity of *Salmonella* strains isolated from poultry carcasses and equipment used in poultry farms in São Paulo. Sereno *et al*. [120] reported similar results, detecting weak and moderate biofilm-forming *Salmonella* strains on frozen poultry carcasses in Paraná. It is important to emphasize that even as a non-biofilm former (10°C after 24 h incubation), *S*. Typhi is the agent of typhoid fever, a disease widely described and clinically characterized by high fever, headache, diarrhea, and abdominal pain after consuming contaminated food [121,122]. Thus, this pathogen poses public health risk because it presents multidrug resistance and resistance genes (*tet(B)*, *tet(C)*, *tet(M)*, and *ampC*) and can attach to surfaces.

## Conclusion

This is the first study to evaluate the biofilm-forming capacity of *Salmonella* spp. isolated from a swine slaughterhouse in Brazil. Furthermore, 21 *E*. *coli* isolates and one *S*. Typhi isolate were detected in the environment and equipment. The *E*. *coli* isolates were multidrug-resistant and harbored resistance and virulence genes. Moreover, 23.8% and 71.4% *E*. *coli* isolates presented strong and moderate biofilm-forming capacity, respectively. The *S*. Typhi isolate was multidrug-resistant and possessed a tetracycline resistance gene. Additionally, it presented moderate biofilm-forming capacity at 37°C after incubating for 72 h. The results of this study suggest a public health risk. The association of the above-stated pathogens with foodborne diseases has been extensively documented, and the decrease in foodborne disease occurrences is closely related to increased food quality through careful hygienic actions within the industries. Reducing or eliminating pathogenic microorganisms before bacterial biofilm formation to ensure the hygienic and sanitary quality of the final product is a guaranteed way to avoid public health risks. Furthermore, it is important to emphasize the risk of spreading resistance genes in the environment. The presence of multiple antimicrobial resistance genes in the isolates in this study indicates the need for the rational use of these drugs to preserve their effectiveness for future use.

## Supporting information

S1 Fig Raw gel image of Fig 1 - PCR confirmation of *Salmonella* Typhi. Row 1) 100-bp marker (Invitrogen®), row 2) negative control, row 3) positive control for *Salmonella* spp., 204-bp fragment (ompC primer), row 4) 204-bp fragment (ompC primer) for *Salmonella* spp., and 738-bp fragment (viaB primer) for Typhi serotype. Visualization on a 2% agarose gel stained with 0.5 μg/mL ethidium bromide in an ultraviolet transilluminator (Major Science®).
(PDF)

S1 Table *E*. *coli* biofilm formation. Biofilm-forming capacity in 21 *E*. *coli* isolates incubated for 24 h and 72 h at three different temperatures (37, 24 and 10°C). The classification is based on the parameters described by Stepanović et al. [62], where ODf is the final optical density of the isolates, and ODn is the negative control optical density. ODn = 0.064 and 0.086 in isolates incubated for 24 h and 0.086 in isolates incubated for 72 h, respecti-vely. The isolates were classified into non-biofilm-forming (NF) when ODf ≤ ODn, weak biofilm-forming (ODn < ODf ≤ 2× ODn), moderate biofilm-forming (2× ODn < ODf ≤ 4× ODn), or strong biofilm-forming (4× ODn < ODf) according to their biofilm-forming ability and intensity.
(PDF)
