# Supplementary material for: Molecular characterization and biofilm-formation analysis of Listeria monocytogenes, Salmonella spp., and Escherichia coli isolated from Brazilian swine slaughterhouses
Source: PLoS One. 2022 Sep 20;17(9):e0274636. doi: 10.1371/journal.pone.0274636 (PMC9488830; doi:10.1371/journal.pone.0274636)

S1 Figure. Raw gel image of Figure 1 - PCR confirmation of *Salmonella* Typhi. Row 1) 100-bp marker (Invitrogen®), row 2) negative control, row 3) positive control for *Salmonella* spp., 204-bp fragment (*ompC* primer), row 4) 204-bp fragment (*ompC* primer) for *Salmonella* spp., and 738-bp fragment (*viaB* primer) for Typhi serotype. Visualization on a 2% agarose gel stained with 0.5 µg/mL ethidium bromide in an ultraviolet transilluminator (Major Science®).

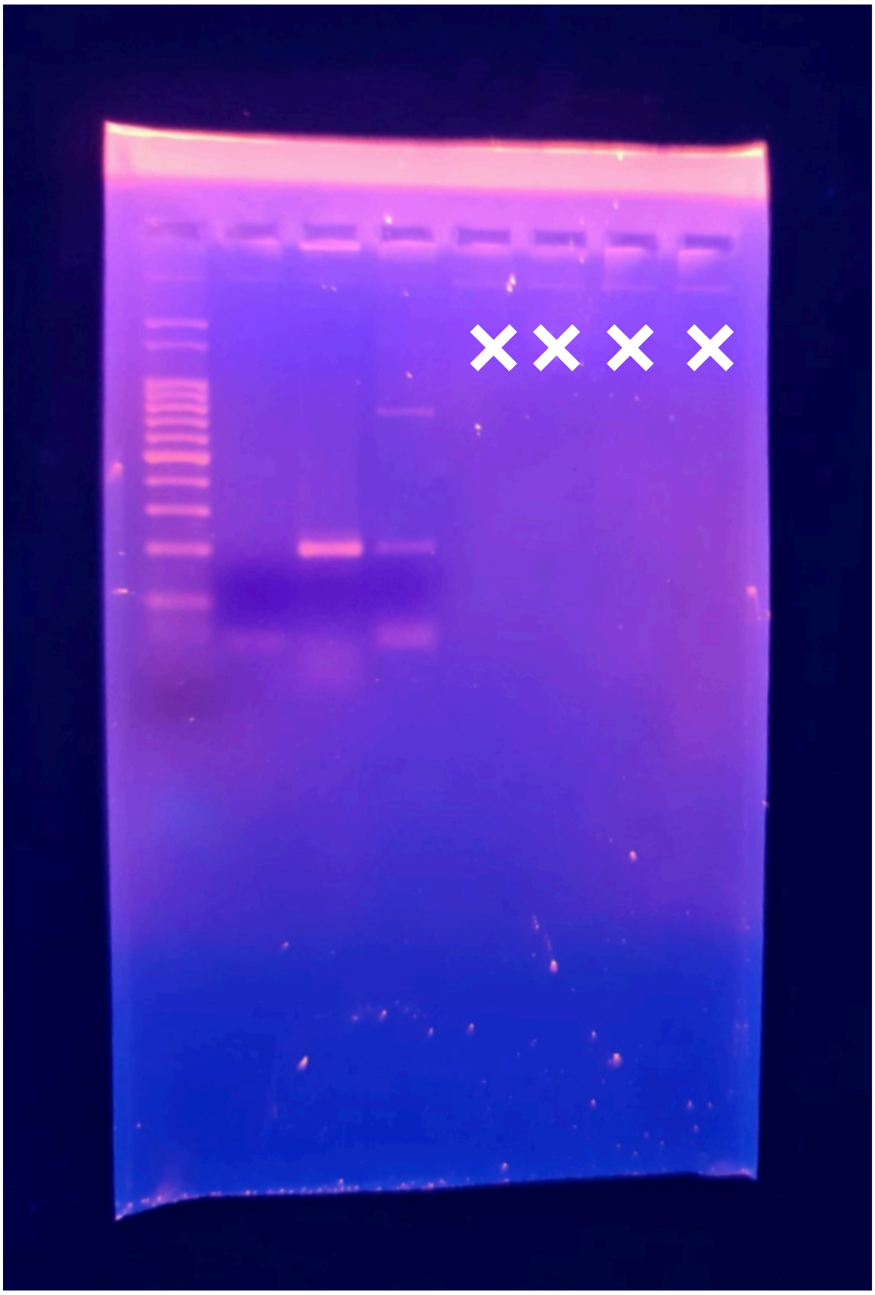

Supplement: S1 Fig — Row 1) 100-bp marker (Invitrogen®), row 2) negative control, row 3) positive control for Salmonella spp., 204-bp fragment (ompC primer), row 4) 204-bp fragment (ompC primer) for Salmonella spp., and 738-bp fragment (viaB primer) for Typhi serotype. Visualization on a 2% agarose gel stained with 0.5 μg/mL ethidium bromide in an ultraviolet transilluminator (Major Science®). (PDF) [file pone.0274636.s001.pdf]
