# Supplementary material for: Molecular characterization and biofilm-formation analysis of Listeria monocytogenes, Salmonella spp., and Escherichia coli isolated from Brazilian swine slaughterhouses
Source: PLoS One. 2022 Sep 20;17(9):e0274636. doi: 10.1371/journal.pone.0274636 (PMC9488830; doi:10.1371/journal.pone.0274636)
Supplement: S1 Table — Biofilm-forming capacity in 21 E. coli isolates incubated for 24 h and 72 h at three different temperatures (37, 24 and 10°C). The classification is based on the parameters described by Stepanović et al. [62], where ODf is the final optical density of the isolates, and ODn is the negative control optical density. ODn = 0.064 and 0.086 in isolates incubated for 24 h and 0.086 in isolates incubated for 72 h, respecti-vely. The isolates were classified into non-biofilm-forming (NF) when ODf ≤ ODn, weak biofilm-forming (ODn < ODf ≤ 2× ODn), moderate biofilm-forming (2× ODn < ODf ≤ 4× ODn), or strong biofilm-forming (4× ODn < ODf) according to their biofilm-forming ability and intensity. (PDF) [file pone.0274636.s002.pdf]

S1 Table. *E. coli* biofilm formation. Biofilm-forming capacity in 21 *E. coli* isolates incubated for 24 h and 72 h at three different temperatures (37, 24 and 10°C).

***E. coli* isolates identification**	**Incubation period**	**ODf at 37 °C**	**ODf at 24 °C**	**ODf at 10 °C**	**Classification at 37 °C**	**Classification at 24 °C**	**Classification at 10 °C**
**1**	24h	0.156	0.188	0.105	weak	moderate	moderate
	72h	0.085	0.095	0.140	weak	weak	weak
**2**	24h	0.064	0.132	0.070	NF	weak	NF
	72h	0.061	0.065	0.087	NF	weak	weak
**5**	24h	0.111	0.124	0.097	weak	weak	weak
	72h	0.099	0.133	0.089	weak	moderate	weak
**6**	24h	0.193	0.192	0.112	moderate	moderate	weak
	72h	0.157	0.134	0.149	moderate	moderate	moderate
**10**	24h	0.193	0.167	0.116	moderate	weak	weak
	72h	0.135	0.133	0.156	moderate	moderate	moderate
**14**	24h	0.110	0.141	0.062	weak	weak	NF
	72h	0.103	0.123	0.063	weak	weak	NF
**15**	24h	0.088	0.125	0.116	weak	weak	weak
	72h	0.087	0.101	0.083	weak	weak	weak
**17**	24h	0.103	0.116	0.111	weak	weak	weak
	72h	0.136	0.117	0.111	moderate	weak	weak
**22**	24h	0.041	0.044	0.043	NF	NF	NF
	72h	0.043	0.044	0.044	NF	NF	NF
**24**	24h	0.068	0.072	0.098	NF	NF	weak
	72h	0.102	0.405	0.313	weak	strong	strong
**25**	24h	0.108	0.094	0.103	weak	weak	weak
	72h	0.114	0.223	0.285	weak	moderate	strong
**26**	24h	0.078	0.090	0.092	NF	weak	weak
	72h	0.118	0.207	0.176	weak	moderate	moderate
**27**	24h	0.069	0.112	0.120	NF	weak	weak
	72h	0.187	0.298	0.191	moderate	strong	moderate
**29**	24h	0.070	0.083	0.075	NF	NF	NF
	72h	0.083	0.188	0.065	weak	moderate	weak

**30**	24h	0.096	0.072	0.122	weak	NF	weak
	72h	0.268	0.165	0.116	strong	moderate	weak
**31**	24h	0.069	0.150	0.179	NF	weak	moderate
	72h	0.078	0.130	0.226	weak	moderate	moderate
**32**	24h	0.072	0.124	0.157	NF	weak	weak
	72h	0.340	0.266	0.312	strong	strong	strong
**33**	24h	0.070	0.094	0.220	NF	weak	moderate
	72h	0.112	0.108	0.174	weak	weak	moderate
**40**	24h	0.248	0.197	0.355	moderate	moderate	strong
	72h	0.201	0.336	0.319	moderate	strong	strong
**41**	24h	0.086	0.076	0.099	weak	weak	weak
	72h	0.131	0.140	0.198	moderate	moderate	moderate
**43**	24h	0.080	0.105	0.221	NF	weak	moderate
	72h	0.084	0.201	0.189	weak	moderate	moderate

* The classification is based on the parameters described by Stepanović *et al.* (2000), where ODf is the final optical density of the isolates, and ODn is the negative control optical density. ODn = 0.064 and 0.086 in isolates incubated for 24 h and 0.086 in isolates incubated for 72 h, respectively. The isolates were classified into non-biofilm-forming (NF) when ODf ≤ ODn, weak biofilm-forming (ODn < ODf ≤ 2× ODn), moderate biofilm-forming (2× ODn < ODf ≤ 4× ODn), or strong biofilm-forming (4× ODn < ODf) according to their biofilm-forming ability and intensity.
